# Complete Genome Analysis of *Paracandidimonas lactea* PBAT-8 Reveals Candidate Functions Associated with PBAT Transformation

**DOI:** 10.3390/toxics14100847

**Published:** 2026-09-23

**Authors:** Xingyu Lin, Yanshuo Pan, Yankun Zhang, Chen Tu, Ying Liu, Yujuan Huang, Shiqi Ma, Dan Wang, Jing Wei, Yongming Luo, Xiaoping Diao

**Affiliations:** 1 Ministry of Education Key Laboratory for Ecology of Tropical Islands, Key Laboratory of Tropical Animal and Plant Ecology of Hainan Province, College of Life Sciences, Hainan Normal University, Haikou 571158, China; 2 State Key Laboratory of Soil & Sustainable Agriculture, Institute of Soil Science, Chinese Academy of Sciences, Nanjing 211135, China; 3 University of Chinese Academy of Sciences, Beijing 100049, China; 4 Nanjing Institute of Environmental Sciences, Ministry of Ecology and Environment, Nanjing 210042, China; 5 State Key Laboratory of Marine Resource Utilization in South China Sea, Hainan University, Haikou 570228, China

**Keywords:** PBAT, biodegradable plastics, environmental fate, microbial transformation, *Paracandidimonas*, complete genome, polyester hydrolase, biodegradation

## Abstract

Poly(butylene adipate-co-terephthalate) (PBAT) is widely used in biodegradable agricultural mulch films, yet its degradation may remain incomplete under field conditions, leading to persistent residues in soil. To investigate microbial processes potentially affecting PBAT fate, we characterized a PBAT-degrading bacterium, *Paracandidimonas lactea* PBAT-8, isolated from long-term plastic-mulched agricultural soil. In minimal salts medium with PBAT as the sole added carbon source, PBAT-8 caused a film mass loss of 6.6% ± 0.14% after 40 days. Hybrid PacBio HiFi and Illumina sequencing generated a complete 4,058,150 bp circular chromosome with a GC content of 61.3% and no detected plasmids. The genome contained 3658 predicted protein-coding genes and encoded functions related to membrane transport, signal transduction, energy metabolism, and the metabolism of aliphatic and aromatic compounds. Six hydrolase-related genes, including alpha/beta hydrolase- and dienelactone hydrolase-family proteins, were prioritized as candidates potentially involved in ester hydrolysis or downstream conversion of PBAT-derived intermediates. Additional genes associated with the transport and metabolism of aliphatic and aromatic compounds were also identified. Although the direct roles of these candidates require experimental validation, the degradation phenotype and complete genome of PBAT-8 provide a genomic basis for further investigation of PBAT transformation in agricultural soils.

## 1. Introduction

The widespread accumulation of conventional non-biodegradable plastics has become a major environmental concern because many widely used synthetic polymers are highly resistant to biological degradation and can persist in terrestrial and aquatic environments for extended periods [1]. During environmental weathering, these plastics may progressively fragment into micro- and nanoplastics, increasing their mobility, bioavailability, and interactions with organisms [2]. Biodegradable polymers have therefore been developed as alternatives for applications in which post-use collection and recycling are difficult or impractical. Agricultural mulching is one such application, because conventional polyethylene (PE) films are often contaminated with soil, damaged during use, and difficult to recover completely after harvest [3].

Poly(butylene adipate-co-terephthalate) (PBAT) is an aliphatic–aromatic copolyester widely used in biodegradable mulch-film formulations because of its flexibility, processability, and biodegradability. Its backbone contains hydrolysable ester linkages connecting flexible aliphatic adipate–butanediol segments with more rigid aromatic terephthalate units [4]. The ester bonds provide sites for enzymatic cleavage, while the flexible aliphatic segments increase chain mobility and accessibility to hydrolytic enzymes [4,5]. These structural features make PBAT more susceptible to enzymatic depolymerization than non-hydrolysable polyolefins, although its degradation rate remains strongly dependent on polymer properties and environmental conditions [5]. Several microorganisms capable of PBAT degradation have been reported. For example, *Bacillus* sp. JO01 achieved approximately 66% PBAT film degradation after 28 days under laboratory conditions [6]. However, degradation efficiencies should be compared cautiously across studies because polymer characteristics, culture conditions, inoculum density, and analytical methods can differ substantially.

However, the designation “biodegradable” does not necessarily indicate rapid or complete degradation under agricultural field conditions [2]. The persistence and transformation of PBAT-based mulch-film residues are influenced by polymer composition, crystallinity, molecular weight, film thickness, surface properties, temperature, moisture, pH, nutrient availability, and the abundance and physiological activity of competent microorganisms. Incomplete degradation may result in the continued presence of visible film fragments, micro- and nanoplastics, or soluble transformation products in soil. Microbial PBAT degradation is expected to involve several sequential and functionally distinct processes, including attachment and biofilm formation on the polymer surface, extracellular or cell-associated depolymerization, transport of soluble oligomers and monomers, and intracellular conversion of aliphatic and aromatic intermediates into central metabolic pathways [7,8]. Identifying microorganisms capable of carrying out these processes and resolving their genetic potential are therefore important for understanding the transformation of PBAT-based biodegradable mulch films in agricultural soils.

Several microbial hydrolase families, particularly cutinases, lipases, carboxylesterases, and other alpha/beta hydrolase-fold enzymes, have been reported to hydrolyze PBAT or structurally related polyesters [9,10]. Nevertheless, the ability to depolymerize a solid polyester cannot be assigned solely from a broad functional annotation. Many alpha/beta hydrolases preferentially act on soluble low-molecular-weight esters, lactones, or intracellular intermediates, whereas carbohydrate esterases classified in the CAZy database primarily describe carbohydrate-associated activities and do not necessarily possess polyester-depolymerizing activity [11,12,13,14]. Candidate enzymes involved in the initial attack on PBAT should therefore be evaluated using multiple lines of evidence, including sequence similarity to experimentally characterized polyester hydrolases, conserved catalytic motifs, predicted subcellular localization, structural features, and genomic context. In addition, PBAT transformation depends not only on initial ester-bond cleavage but also on cellular functions involved in surface attachment, substrate sensing, transport, stress adaptation, and the downstream metabolism of released aliphatic and aromatic compounds. Of particular relevance are enzymes involved in the activation and ring cleavage of aromatic intermediates, which may facilitate the assimilation of compounds derived from the terephthalate-containing fraction of PBAT.

Given the limited number of well-characterized PBAT-degrading bacteria, screening plastic-impacted environments for microorganisms capable of PBAT transformation is important for expanding the known taxonomic and functional diversity of PBAT-associated degraders [5,6]. Members of the genus *Paracandidimonas* are Gram-negative bacteria belonging to the family Alcaligenaceae and have been recovered from soils, wastewater, and other environments affected by organic contaminants [15]. Some members of this genus exhibit broad metabolic versatility toward structurally diverse carbon compounds; however, their potential roles in the transformation of biodegradable polyesters remain insufficiently characterized. In the present study, strain PBAT-8 was isolated from agricultural soil subjected to long-term plastic mulching and exhibited PBAT film-transformation activity during preliminary screening. This environmental origin and transformation phenotype prompted further investigation of its genomic characteristics and potential functions associated with PBAT transformation.

Accordingly, this study aimed to (i) determine the taxonomic characteristics of strain PBAT-8; (ii) obtain and annotate its complete genome using PacBio HiFi and Illumina sequencing; (iii) characterize genomic functions potentially associated with PBAT surface interaction, ester-bond hydrolysis, transport, and the downstream metabolism of aliphatic and aromatic degradation products; and (iv) prioritize candidate hydrolase-related genes for subsequent functional validation. Particular attention was given to distinguishing proteins potentially involved in extracellular or cell-associated polymer depolymerization from enzymes more likely to participate in the intracellular conversion of soluble oligomers and aromatic intermediates. By linking a verified PBAT-transformation phenotype with complete genome information, this study provides a genome-resolved basis for understanding microbial processes that may influence the transformation of PBAT-based biodegradable mulch-film residues in agricultural soils.

## 2. Materials and Methods

### 2.1. Bacterial Strain, Isolation Source, and Routine Cultivation

*Paracandidimonas lactea* PBAT-8 was isolated in our laboratory from agricultural soil collected on 2 September 2020, from a vegetable field in Dongying, Shandong, China (37°45′16.96″ N, 118°58′19.86″ E). The field had been continuously mulched with conventional plastic films for over 5 years, and visible residual film fragments were present at the time of sampling. Soil was collected from the 0–15 cm topsoil layer using a sterile soil auger according to a five-point composite sampling procedure. The samples were thoroughly mixed, sieved through a 2 mm mesh, stored at 4 °C, and processed within 48 h.

Candidate PBAT-transforming strains were obtained through enrichment and screening under minimal-medium conditions with PBAT as the only externally added carbon source. Briefly, the soil sample was added to minimal salt medium (MSM, pH 7.0) containing 0.7 g L^−1^ K_2_HPO_4_·3H_2_O, 0.7 g L^−1^ KH_2_PO_4_, 1.0 g L^−1^ NH_4_NO_3_, 0.5 g L^−1^ NaCl, 0.5 g L^−1^ MgSO_4_·7H_2_O, 0.002 g L^−1^ FeSO_4_·7H_2_O, 0.002 g L^−1^ ZnSO_4_·7H_2_O, and 0.001 g L^−1^ MnSO_4_·H_2_O, together with sterile PBAT film pieces. The cultures were incubated at 30 °C with shaking at 180 rpm for 3 months. An aliquot of the enrichment culture was then transferred to fresh MSM containing sterile PBAT film as the only externally added carbon source and further incubated. The enrichment culture was subsequently serially diluted and spread onto minimal salt agar plates overlaid with sterile PBAT films, followed by incubation at 30 °C. Colonies growing beneath the PBAT films were picked and purified on nutrient agar.

Nine candidate strains were obtained through this procedure and subsequently evaluated for their effects on PBAT films. Strain PBAT-8 exhibited the highest PBAT film weight-loss rate among the candidate isolates and was therefore selected for further phenotypic and genomic characterization. For routine cultivation and colony observation, strain PBAT-8 was streaked onto beef extract peptone (BMP) agar containing 3.0 g L^−1^ beef extract, 10.0 g L^−1^ peptone, 5.0 g L^−1^ NaCl, and 15 g L^−1^ agar. The medium pH was adjusted to 7.0 before autoclaving at 121 °C for 20 min. The inoculated plates were incubated at 37 °C for 48 h. Strain PBAT-8 has been deposited in the China Center for Type Culture Collection (CCTCC) under accession number CCTCC M 20221378 [16].

### 2.2. Verification of the PBAT Film Transformation Phenotype

PBAT film was obtained from Shandong Qingtian Plastic Industry Co., Ltd., Zibo, China. The film had a thickness of 15 μm and was cut into 5 cm × 5 cm pieces. Before incubation, the PBAT film pieces were immersed in 75% (*v*/*v*) ethanol for 2 h for surface disinfection, rinsed three times with sterile ultrapure water to remove residual ethanol, and dried at 50 °C under sterile conditions to constant weight before determination of the initial dry mass.

The PBAT film transformation assay was performed in 250 mL Erlenmeyer flasks containing 45 mL of MSM. Three pre-weighed PBAT film pieces were added to each flask. A single colony of strain PBAT-8 was inoculated into BMP broth and cultured at 37 °C until sufficient biomass was obtained. The cells were harvested by centrifugation, washed twice with sterile phosphate-buffered saline (PBS), and resuspended in sterile MSM. The cell suspension was adjusted to an OD_600_ of 1.0, and 5 mL was inoculated into each flask. Uninoculated flasks containing the same medium and PBAT film were included as abiotic controls. Each treatment consisted of three independent replicate flasks (*n* = 3), which were treated as biological replicates. All flasks were incubated at 30 °C with shaking at 180 rpm for 40 days. For the prolonged-incubation experiment, a parallel set of flasks was incubated under the same conditions for 180 days.

At the end of incubation, the PBAT films were recovered and washed using 2% (*w*/*v*) sodium dodecyl sulfate (SDS) at 180 rpm for 4 h to remove attached biomass and residual medium. The films were then rinsed thoroughly with ultrapure water, dried at 50 °C to constant weight, and weighed to determine the final dry mass. PBAT film mass loss was calculated as follows:

Mass loss (%) = [(*m*_0_ − *m_t_*)/*m*_0_] × 100

where *m*_0_ and *m_t_* represent the initial and final dry masses of the PBAT film, respectively.

### 2.3. Colony and Cellular Morphology

Colony morphology was recorded after 48 h of incubation on BMP agar. Colony diameter, shape, margin, elevation, color, opacity, surface texture, and moisture were documented. For scanning electron microscopy (SEM), bacterial cells and PBAT film samples were prepared as follows. Bacterial cells were harvested from liquid culture by centrifugation at 8000 rpm for 5 min and washed three times with sterile 0.1 mol L^−1^ phosphate-buffered saline (PBS). PBAT film samples were recovered from both the PBAT-8-inoculated treatment and the corresponding uninoculated control after 40 and 180 days of incubation. Both bacterial cells and PBAT film samples were subsequently fixed in 2.5% glutaraldehyde for 12 h according to a previously described procedure with modifications [17], washed three times with sterile 0.1 mol L^−1^ PBS, and sequentially dehydrated in 10%, 30%, 50%, 70%, and 90% ethanol for 10 min at each concentration, followed by two 15-min treatments with 100% ethanol. The dehydrated samples were air-dried at room temperature, mounted on SEM stubs using conductive carbon tape, sputter-coated with gold, and examined using a Hitachi S-4800 scanning electron microscope (Hitachi High-Technologies Corporation, Tokyo, Japan) at an accelerating voltage of 3.0 kV. PBAT films from both PBAT-8-inoculated and uninoculated control treatments were examined using this procedure at the corresponding incubation times. SEM images of bacterial cells were used for qualitative characterization of the overall cell morphology of strain PBAT-8, whereas SEM images of PBAT films were used to assess surface alterations after incubation.

### 2.4. Genomic DNA Extraction and Quality Assessment

A single colony of strain PBAT-8 was inoculated into 200 mL of BMP broth and cultured at 37 °C with shaking at 180 rpm for 12 h. Cells were harvested by centrifugation at 12,000× *g* for 10 min. Genomic DNA was extracted following a previously described procedure with modifications [18]. Briefly, approximately 50 mg of wet cell biomass was resuspended in 1 mL of lysis buffer containing 20 mg mL^−1^ lysozyme and 10 mmol L^−1^ Tris-HCl (pH 8.0) and incubated at 37 °C for 30 min. Subsequently, 20 μL of Proteinase K (20 mg mL^−1^) and 100 μL of 10% (*w*/*v*) SDS were added, and the suspension was incubated at 56 °C for 2 h.

Genomic DNA was extracted with phenol–chloroform–isoamyl alcohol (25:24:1, *v*/*v*/*v*). The aqueous phase was recovered and mixed with 0.1 volume of 3 mol L^−1^ sodium acetate (pH 5.2) and two volumes of absolute ethanol, followed by incubation at −20 °C for 30 min to precipitate DNA. The precipitated DNA was washed twice with 70% ethanol, air-dried, and dissolved in 50 μL of TE buffer.

DNA concentration and purity were measured with a NanoDrop 2000 spectrophotometer (Thermo Fisher Scientific, Wilmington, DE, USA), and DNA integrity was evaluated by electrophoresis on a 1% agarose gel at 100 V for 30 min. DNA samples with an A_260_/A_280_ ratio of 1.8–2.0 and an intact high-molecular-weight band were used for library construction.

### 2.5. Library Construction and Genome Sequencing

Illumina short-read and PacBio HiFi libraries were prepared from the same high-quality genomic DNA preparation. For Illumina sequencing, genomic DNA was fragmented to approximately 400–500 bp using a Covaris M220 focused ultrasonicator (Covaris, Inc., Woburn, MA, USA). A paired-end library was constructed using the NEXTFLEX Rapid DNA-Seq Kit (Revvity, Waltham, MA, USA), including end repair, 5′ phosphorylation, 3′ adenylation, adaptor ligation, and PCR enrichment. The library was sequenced on an Illumina NovaSeq 6000 platform using 2 × 150 bp paired-end sequencing.

For PacBio sequencing, high-molecular-weight genomic DNA was fragmented to an average size of approximately 10 kb. The fragmented DNA was subjected to damage repair, end repair, and SMRTbell-adaptor ligation using the SMRTbell Prep Kit 3.0 (Pacific Biosciences, Menlo Park, CA, USA). The resulting library was sequenced on a PacBio Sequel IIe platform using one SMRT Cell to generate circular consensus sequencing (CCS) HiFi reads [19,20].

### 2.6. Genome Assembly and Quality Assessment

Bioinformatic analyses were performed using the Majorbio Cloud Platform (Majorbio Bio-Pharm Technology Co., Ltd., Shanghai, China). Illumina reads were filtered and trimmed using fastp v0.23.0 to remove adaptor sequences and low-quality reads [21]. The PacBio HiFi reads and quality-filtered Illumina reads were subjected to hybrid assembly using Unicycler v0.4.8 [22]. The resulting assembly was polished using Pilon v1.22 with the Illumina reads to correct residual small insertions, deletions, and nucleotide substitutions [23]. Genome completeness, contamination, and strain heterogeneity were assessed using CheckM v1.1.3. The assembled genome showed 100% completeness, 0.03% contamination, and no detectable strain heterogeneity. The complete genome sequence was deposited in GenBank under accession number PRJNA1497611.

### 2.7. Genome-Based Taxonomic Identification

The 16S rRNA gene sequence of strain PBAT-8 was extracted from the complete genome and compared with reference sequences in the NCBI database using BLAST v[2.17.0+]. Closely related type strains were subsequently selected for genome-level taxonomic comparison. Whole-genome average nucleotide identity (ANI) was calculated using FastANI [24], and digital DNA–DNA hybridization (dDDH) values were estimated using the Genome-to-Genome Distance Calculator (GGDC) [25].

### 2.8. Gene Prediction and Functional Annotation

Protein-coding sequences were predicted using Prodigal v2.6.3 [26], with Glimmer [27] and GeneMarkS [28] used as complementary gene-prediction tools. Transfer RNA genes were identified using tRNAscan-SE v2.0 [29], and ribosomal RNA genes were identified using Barrnap v0.9. Predicted proteins were functionally annotated by comparison against the NCBI non-redundant protein database (NR), Swiss-Prot, Pfam, Gene Ontology (GO) [30,31], Clusters of Orthologous Groups (COG) [32,33], and Kyoto Encyclopedia of Genes and Genomes (KEGG) [34,35]. Sequence-similarity searches were conducted using DIAMOND [36] or BLAST, whereas profile hidden Markov model searches were performed using HMMER [37], as appropriate for each database. An E-value threshold of 1 × 10^−5^ was applied to sequence-similarity searches unless a database-specific trusted cutoff was available.

Functional assignments were made based on the best-supported matches after considering E-value, sequence identity, alignment coverage, conserved domains, agreement among databases, and available Enzyme Commission (EC) number assignments. Carbohydrate-active enzymes were annotated against the CAZy database [11,12,13,14]. Because PBAT is a polyester rather than a carbohydrate polymer, CAZyme annotations were treated as general functional classifications and were not considered direct evidence of PBAT-depolymerizing activity.

### 2.9. Analysis of Candidate Hydrolases-Related Proteins and Aromatic-Compound Metabolism

Candidate proteins potentially associated with PBAT transformation were screened by integrating functional annotations from NR, Swiss-Prot, Pfam, KEGG databases, together with available EC number assignments. Proteins annotated as hydrolase, esterase, or related enzyme families by multiple annotation sources were further examined for conserved domains, putative catalytic motifs, and predicted subcellular localization.

Subcellular localization was predicted using PSORTb, and N-terminal signal peptides were predicted using SignalP. Based on consistency among functional annotations and the presence of hydrolase-related domains or putative catalytic features, six candidate hydrolase-related proteins encoded by gene0190, gene0673, gene1155, gene3555, gene3558, and gene0564 were prioritized for further sequence and phylogenetic analyses.

Homologous reference proteins were retrieved from NCBI and Swiss-Prot using BLASTp. Protein sequences were aligned using MAFFT [38], and maximum-likelihood phylogenetic trees were constructed using IQ-TREE, with the best-fit substitution model selected automatically and branch support evaluated using 1000 bootstrap replicates.

Genes potentially involved in the transport and downstream metabolism of aromatic PBAT-derived intermediates were identified based on KEGG annotations and previously characterized aromatic-compound degradation pathways [39,40,41]. Dienelactone hydrolase-family proteins were interpreted as candidate enzymes potentially involved in downstream metabolism rather than as direct PBAT depolymerases.

### 2.10. Statistical Analysis

PBAT film mass-loss data are presented as the mean ± standard deviation (SD) of three independent replicate flasks. Statistical comparisons between the PBAT-8 treatment and the uninoculated control were performed using an independent-samples *t*-test, with *p* < 0.05 considered statistically significant.

## 3. Results

### 3.1. Morphological Characteristics of Strain PBAT-8 and PBAT Film Transformation

Following enrichment and isolation, nine candidate strains were obtained and subsequently screened for their effects on PBAT films in liquid minimal salt medium. Among these isolates, strain PBAT-8 exhibited the highest PBAT film mass-loss rate and was therefore selected for further phenotypic and genomic characterization. After 48 h of cultivation on BMP agar, strain PBAT-8 formed small, circular to nearly circular colonies with regular margins. The colonies were smooth, moist, slightly glossy, and cream-white to pale cream-yellow (Figure 1A). Scanning electron microscopy showed predominantly short rod-shaped cells with rounded ends, approximately 1–2 μm in length (Figure 1B). In the endpoint assay, PBAT films incubated with strain PBAT-8 showed a mass loss of 6.6% ± 0.14% after 40 days, while the uninoculated control showed an approximately 5.1% ± 0.10% decrease in film mass over the same period. After prolonged incubation for 180 days, PBAT film mass loss reached 21.7% ± 3.1% in the PBAT-8 treatment and 7.8% ± 1.6% in the uninoculated control (mean ± SD, *n* = 3), with representative SEM images of the corresponding film surfaces shown in Figure S1.

### 3.2. Genome Assembly and General Genomic Features of Strain PBAT-8

After quality filtering, 7,214,614 Illumina paired-end clean reads (1.086 Gb) were obtained, corresponding to approximately 267.7-fold genome coverage. PacBio sequencing generated 34,739 HiFi reads totaling 272.8 Mb, with an average read length of 7852 bp and a read N50 of 8060 bp, corresponding to approximately 67.22-fold genome coverage. Hybrid assembly of the PacBio HiFi and Illumina reads generated a single circular chromosome of 4,058,150 bp, and no plasmid was detected (Table 1 and Figure 2). CheckM analysis indicated 100% genome completeness and 0.03% contamination, with no detected strain heterogeneity. K-mer-based genome coverage was 96.5%, whereas read-mapping coverage reached 100% for both the Illumina and PacBio datasets. The chromosome had a GC content of 61.30%.

A total of 3658 protein-coding genes were predicted, with a combined length of 3,656,508 bp and an average gene length of 999.59 bp (Table 2). The gene density was 0.90 genes kb^−1^, and protein-coding regions accounted for 90.10% of the chromosome. The GC content was 61.77% in coding regions and 56.98% in intergenic regions. The genome also contained nine rRNA genes, 55 tRNA genes, and 21 small RNA genes [42].

### 3.3. Taxonomic Identification and Functional Annotation of the PBAT-8 Genome

To determine the taxonomic position of strain PBAT-8, its complete genome sequence was compared with type-strain genomes available in the NCBI RefSeq database using average nucleotide identity (ANI) analysis. Strain PBAT-8 showed 99.836% ANI with the type strain genome of *Paracandidimonas lactea*, with 93.9% genome coverage. The corresponding genome-to-genome distance analysis yielded a digital DNA–DNA hybridization (dDDH) value of 98.9%. Both values substantially exceeded the commonly accepted species-level thresholds of approximately 95–96% for ANI and 70% for dDDH, supporting the assignment of strain PBAT-8 to *Paracandidimonas lactea*. Accordingly, the strain is formally designated as *Paracandidimonas lactea* PBAT-8.

Most predicted proteins received at least one functional assignment. NR provided the highest annotation coverage, with 3619 genes assigned, followed by Pfam (3348 genes), COG (3294), KEGG (3105), Swiss-Prot (3049), and GO (2133).

COG classification showed that amino acid transport and metabolism was the most abundant category (430 genes), followed by energy production and conversion (337 genes), lipid transport and metabolism (302 genes), and transcription (297 genes) (Figure 3). Genes assigned to inorganic-ion transport, translation, cell-wall and membrane biogenesis, carbohydrate metabolism, coenzyme metabolism, signal transduction, and post-translational modification were also represented.

GO annotations were distributed among the biological-process, cellular-component, and molecular-function domains. Major biological-process terms included transmembrane transport, transcriptional regulation, translation, proteolysis, phosphorylation, carbohydrate metabolism, and cell-wall organization. Cellular-component annotations were dominated by the cytoplasm, plasma membrane, periplasmic space, outer membrane, ribosome, and ATP-binding cassette transporter complexes, whereas major molecular-function terms included ATP binding, transmembrane transporter activity, hydrolase activity, oxidoreductase activity, transferase activity, and metal-ion binding.

KEGG annotation was dominated by metabolism and environmental information processing (Figure 4). In addition to pathways related to amino acid, carbohydrate, energy, lipid, and cofactor metabolism, 111 genes were assigned to xenobiotic biodegradation and metabolism. Environmental-information-processing functions included 302 genes associated with signal transduction and 224 genes associated with membrane transport. Genes related to cellular-community functions and motility were also identified.

CAZy annotation identified 94 genes across six CAZyme classes. Glycosyltransferases (GTs) were the most abundant class (34 genes), followed by glycoside hydrolases (GHs; 20), auxiliary activity enzymes (AAs; 19), carbohydrate esterases (CEs; 19), carbohydrate-binding modules (CBMs; 1), and polysaccharide lyases (PLs; 1). Together, GTs, GHs, AAs, and CEs accounted for 92 of the 94 CAZyme-annotated genes, indicating that these four classes dominated the CAZyme repertoire of strain PBAT-8.

### 3.4. Candidate Hydrolases and Aromatic-Compound Metabolism

Six hydrolase-related genes—gene0190, gene0673, gene1155, gene3555, gene3558, and gene0564—were selected for further analysis (Table 3). Gene0190, gene0673, gene3555, and gene3558 were annotated as alpha/beta hydrolase-fold proteins, gene1155 as a distinct alpha/beta-fold hydrolase, and gene0564 as a dienelactone hydrolase-family protein. Several candidates carried EC 3.1.1.24 assignments; gene3555 was additionally assigned EC 4.1.1.44, whereas gene1155 was assigned EC 3.5.1.106. Phylogenetic analysis placed the six PBAT-8 proteins at separate positions among their homologous reference sequences (Figure 5). Genome annotation also identified genes associated with the transport and metabolism of aromatic compounds, including functions assigned to aromatic-ring activation, cleavage, and downstream conversion.

## 4. Discussion

### 4.1. PBAT Transformation Phenotype and Environmental Relevance

Strain PBAT-8 caused a PBAT film mass loss of 6.6% after 40 days, indicating a measurable capacity for PBAT transformation under the tested conditions. PBAT degradation generally begins with hydrolytic cleavage of ester bonds, followed by the uptake and metabolism of soluble oligomers and monomers [3,4]. The activity of PBAT-8 was lower than that reported for some previously described PBAT-degrading bacteria. For example, *Bacillus* sp. JO01 achieved substantially greater PBAT film degradation under different culture conditions [6]. Other bacterial strains and microbial polyester hydrolases have also shown different levels of activity toward PBAT [9]. However, degradation efficiencies should be compared with caution, because polymer composition, film thickness, crystallinity, inoculum density, incubation conditions, and analytical methods can strongly affect the measured degradation rate [3,4]. The 6.6% mass loss observed here therefore represents partial PBAT transformation, rather than evidence of complete biodegradation or mineralization. Nevertheless, this phenotype provides an experimental basis for examining the genomic traits potentially associated with PBAT transformation in strain PBAT-8.

PBAT-8 was isolated from agricultural soil subjected to long-term plastic mulching, which represents an environmentally relevant source for screening microorganisms associated with plastic transformation. Repeated plastic inputs can alter soil microhabitats and provide polymer surfaces and plastic-derived compounds that may influence microbial colonization and substrate utilization [6,7,8]. However, the historical mulch films at the sampling site were not confirmed to contain PBAT, so the isolation source does not demonstrate specific adaptation of PBAT-8 to this polymer. The significance of this strain therefore lies primarily in its experimentally observed PBAT-transformation phenotype and its taxonomic identity within *Paracandidimonas*. Reports of PBAT transformation have involved bacteria from several phylogenetic groups, suggesting that this capacity is not restricted to a single bacterial lineage [5]. PBAT-8 thus expands the range of bacterial taxa associated with PBAT transformation and provides a useful genomic resource for investigating the functions underlying this phenotype.

### 4.2. Genome Features Relevant to PBAT Transformation

The complete chromosome of PBAT-8 provides a reliable genomic basis for examining its metabolic potential and candidate functions associated with PBAT transformation. No plasmids were detected, indicating that the candidate functions discussed here are chromosome encoded. The relatively high GC content represents a genomic characteristic of PBAT-8, but does not imply an enhanced capacity for PBAT degradation [43,44]. More importantly, the genome contains diverse functions related to membrane transport, signal transduction, motility, energy metabolism, and xenobiotic metabolism. These functions are common in metabolically versatile environmental bacteria and may support responses to chemically diverse substrates [5,6,7]. They should therefore be viewed as background physiological capacities, rather than as PBAT-specific traits.

Membrane transport and signal-transduction systems may become important when soluble compounds are released during polymer transformation. ABC transporters mediate the uptake and export of diverse substrates, while two-component systems allow bacteria to sense environmental changes and regulate cellular responses [45,46]. Motility and energy-metabolism functions may further support substrate access and cellular activity. In PBAT-8, these broad physiological functions occur together with genes related to ester hydrolysis and aromatic-compound metabolism. This combination provides a metabolic framework for processing chemically diverse organic compounds, including potential intermediates generated during PBAT transformation.

### 4.3. CAZyme Repertoire and Its Interpretive Limits

The CAZyme repertoire of PBAT-8 reflects a broad enzymatic capacity for carbohydrate processing and ester-related reactions. Glycosyltransferases, glycoside hydrolases, and auxiliary-activity enzymes mainly participate in the synthesis, modification, and degradation of carbohydrate-derived compounds [11,12,13,14]. These functions may contribute to the utilization of diverse organic substrates and therefore reflect the general metabolic versatility of PBAT-8. However, they are not specific indicators of PBAT degradation. Carbohydrate esterases are of greater interest in this context because PBAT contains hydrolysable ester bonds. Nevertheless, CAZy-classified carbohydrate esterases are primarily characterized by their activity toward ester substitutions in carbohydrate-associated substrates, so their annotation does not directly imply activity toward PBAT.

The 19 carbohydrate esterase genes identified in PBAT-8 nevertheless provide a useful pool of ester-active candidates for further investigation. Proteins within the same esterase family can differ substantially in substrate specificity, making functional annotation alone insufficient to assign polyester-hydrolyzing activity [47]. Their potential relevance to PBAT transformation therefore depends on additional features, including catalytic motifs, substrate accessibility, and cellular localization. Overall, the CAZyme profile is best viewed as complementary information describing the enzymatic background of PBAT-8, rather than evidence for a dedicated PBAT-depolymerizing system. This genomic context also provides a basis for the subsequent evaluation of individual hydrolase-related proteins with more specific potential roles in PBAT transformation.

### 4.4. Candidate Hydrolases and PBAT-Derived Intermediates

Experimentally characterized PBAT-active enzymes mainly include cutinases, carboxylesterases, and related polyester hydrolases, several of which can directly cleave ester bonds in the polymer backbone [5,9]. The six hydrolase-related proteins identified in PBAT-8 represent a functionally diverse set of candidates rather than a single PBAT-depolymerase group. Their positions in the phylogenetic analysis also suggest substantial sequence and functional differentiation. These proteins therefore provide targets for evaluating ester-related functions in PBAT-8, but their possible roles should be considered according to their predicted biochemical properties and cellular context.

Genome-based studies of PBAT-transforming bacteria remain relatively limited, but several reported strains provide useful points of comparison. Whole-genome analysis of the bioplastic-degrading *Bacillus* sp. JY35, which is capable of PBAT degradation, identified three carboxylesterases and one triacylglycerol lipase as candidate enzymes, with subsequent functional screening demonstrating bioplastic-degrading activity of one prioritized esterase [48]. In *Roseibium aggregatum* ZY-1, genome analysis identified putative PETase, carboxylesterases, and an arylesterase, together with genes associated with aromatic-compound metabolism, including *pobA*, *pca*, *dca*, and *paa* pathway components [49]. These observations suggest that PBAT-transforming bacteria may combine ester-cleaving enzymes with metabolic functions supporting the subsequent utilization of polymer-derived products. PBAT-8 shows a broadly similar functional pattern, with several alpha/beta hydrolase-related candidates together with genes associated with aromatic-compound transport, activation, and ring cleavage. However, whether the six PBAT-8 candidates are homologous to, or perform functions comparable to, these previously reported enzymes remains unresolved.

Several candidates appear more consistent with downstream metabolism than with the initial depolymerization of intact PBAT. Proteins assigned to EC 3.1.1.24 or EC 4.1.1.44, together with the dienelactone hydrolase-family protein gene0564, are associated with lactone-processing reactions in aromatic-compound degradation pathways [39,40,41]. The genome also contains genes involved in aromatic-compound transport, ring activation, cleavage, and subsequent metabolism. Together, these functions indicate a metabolic capacity for processing aromatic intermediates after their release or formation. However, this genomic capacity does not establish a complete terephthalate-assimilation pathway, because the connection between PBAT-derived terephthalate and these annotated reactions has not been experimentally demonstrated.

The phenotypic and genomic results therefore provide complementary, but distinct, levels of evidence. The observed film mass loss supports partial PBAT transformation under the tested conditions, whereas the genome encodes several candidate hydrolase-related proteins that may participate in initial PBAT hydrolysis, as well as functions potentially involved in the transport and downstream metabolism of PBAT-derived intermediates. The localization and direct activity of these candidate hydrolases toward intact PBAT remain unverified. Candidate proteins with appropriate catalytic motifs and extracellular or membrane-associated localization are therefore the most relevant targets for subsequent biochemical and genetic validation. From a broader applied perspective, polyester hydrolases, including engineered cutinases, have been investigated for PBAT depolymerization and recovery of monomeric products, highlighting their potential for enzymatic recycling PBAT-containing waste [4,10]. However, whether PBAT-8 or its candidate hydrolases have comparable activity or application potential remains unknown and requires direct functional and process-level evaluation.

### 4.5. Study Limitations and Future Directions

Several limitations constrain the mechanistic interpretation of the present study. Although PBAT film mass loss was assessed after 40 and 180 days, PBAT transformation was examined under only one temperature and agitation condition, while changes in polymer molecular weight, surface chemistry, degradation products, carbon assimilation, and mineralization were not measured. Functional assignments of the candidate genes are based primarily on sequence annotation and phylogenetic analysis rather than expression, enzymatic, or genetic evidence. Moreover, comparative genomic analysis with closely related *Paracandidimonas* strains was not performed. Therefore, it remains unclear whether the six candidate hydrolase-related genes identified in PBAT-8 are specific or are broadly conserved within the genus *Paracandidimonas*. Comparative analyses of closely related genomes will be required to clarify the taxonomic distribution and potential specificity of these candidate functions. The substrate range of PBAT-8 also remains unknown, and its ability to transform other structurally related biodegradable polyesters should be experimentally evaluated. Transcriptomic and proteomic analyses under different polymer substrates may further help identify substrate-responsive genes and enzymes. Within these limitations, the complete genome of PBAT-8 provides a useful resource for further investigation of the molecular and metabolic processes associated with PBAT transformation.

## 5. Conclusions

*Paracandidimonas lactea* PBAT-8, isolated from long-term plastic-mulched agricultural soil, caused a 6.6% mass loss of PBAT film after 40 days. Its complete 4.06-Mb genome contained 3658 protein-coding genes and several candidate functions related to ester hydrolysis, transport, and aromatic-intermediate metabolism. These findings support a multistage PBAT transformation process, although direct PBAT depolymerase activity and complete mineralization remain to be verified. Strain PBAT-8 therefore provides a genomic basis for understanding the transformation of PBAT-based mulch-film residues in agricultural soils.

## Figures and Tables

**Figure 1:**
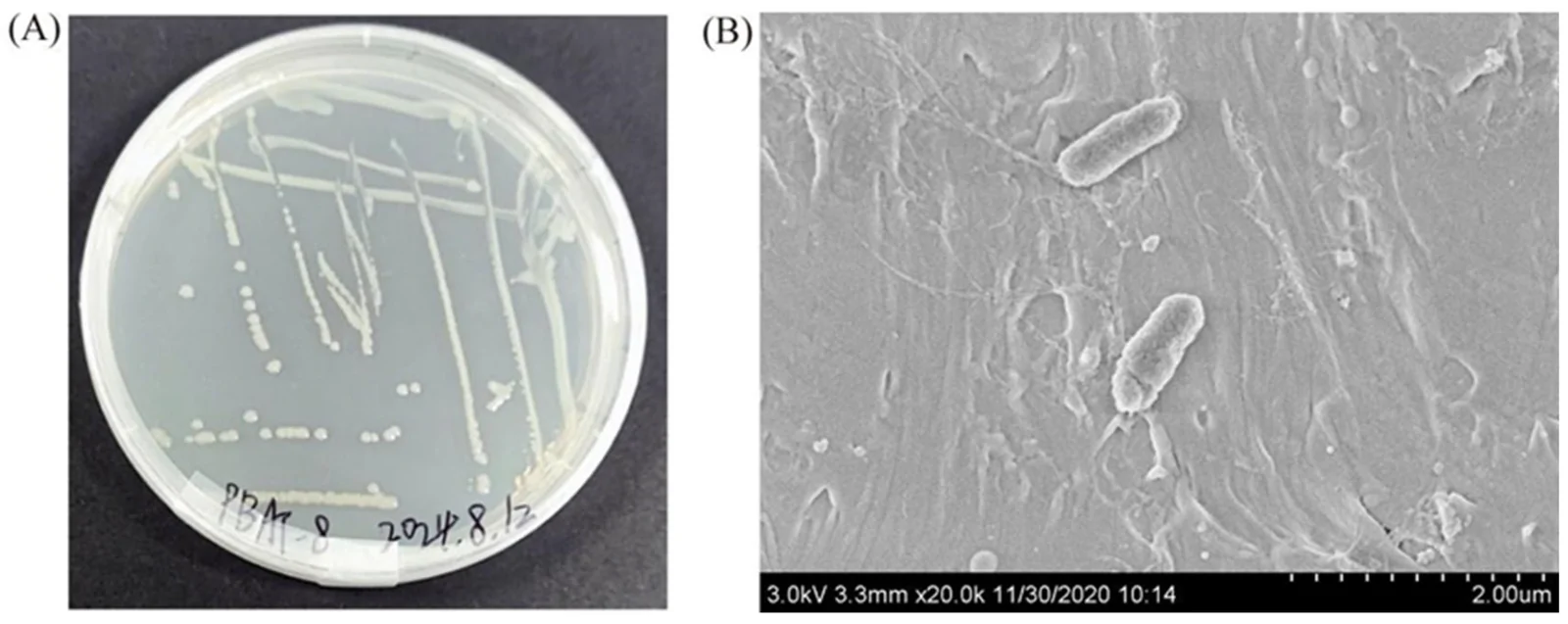
Colony and cellular morphology of *Paracandidimonas lactea* PBAT-8. (**A**) Colony appearance on beef extract–peptone agar after 48 h of incubation. (**B**) Scanning electron micrograph of PBAT-8 cells showing a short rod-shaped morphology. Scale bar, 2 μm.

**Figure 2:**
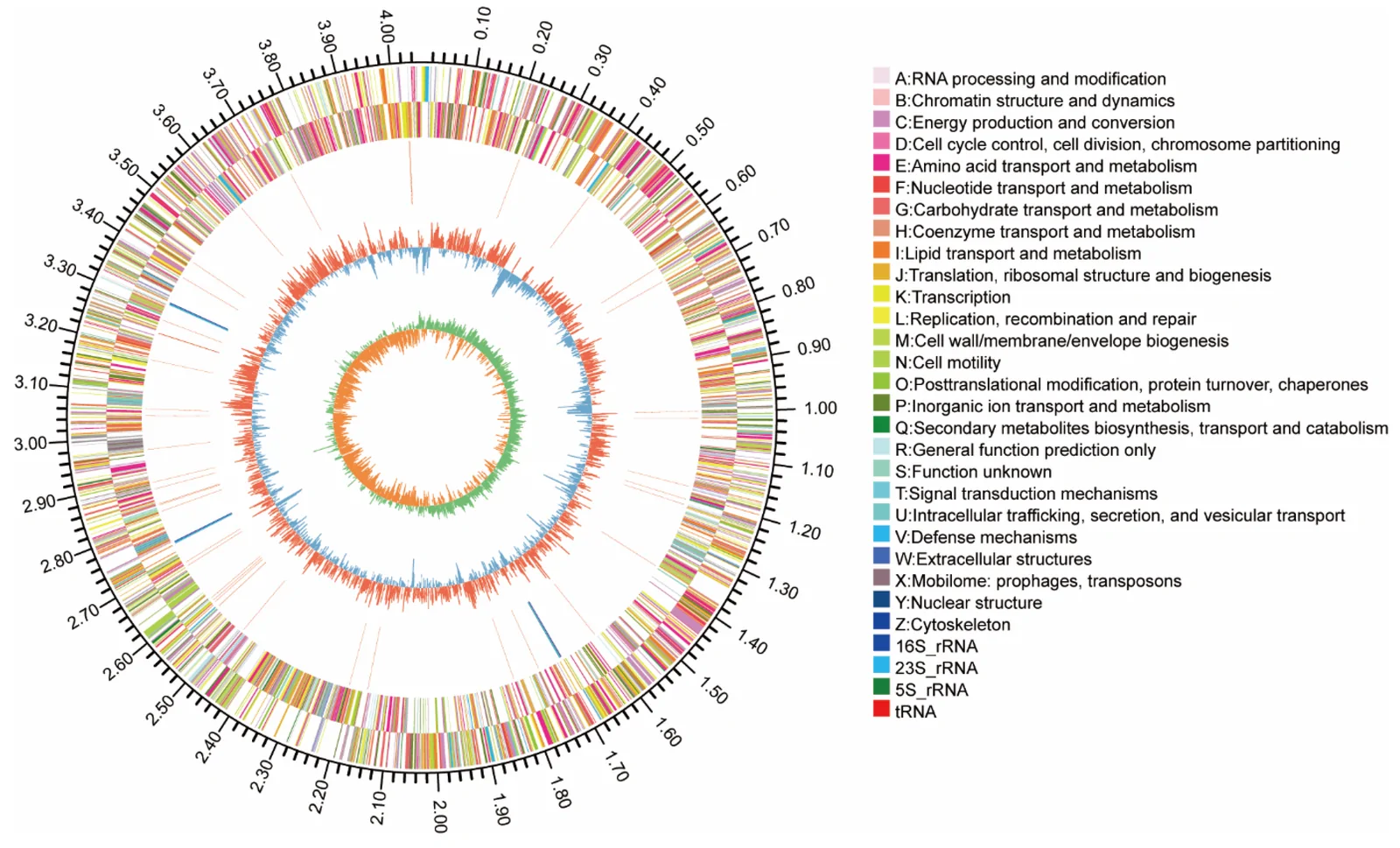
Circular genome map of *Paracandidimonas lactea* PBAT-8. The outer scale indicates genome position. The outer colored tracks show coding sequences on the forward and reverse strands classified by COG category and the positions of RNA genes. Inner tracks show GC content and GC skew (green, positive values; orange, negative values).

**Figure 3:**
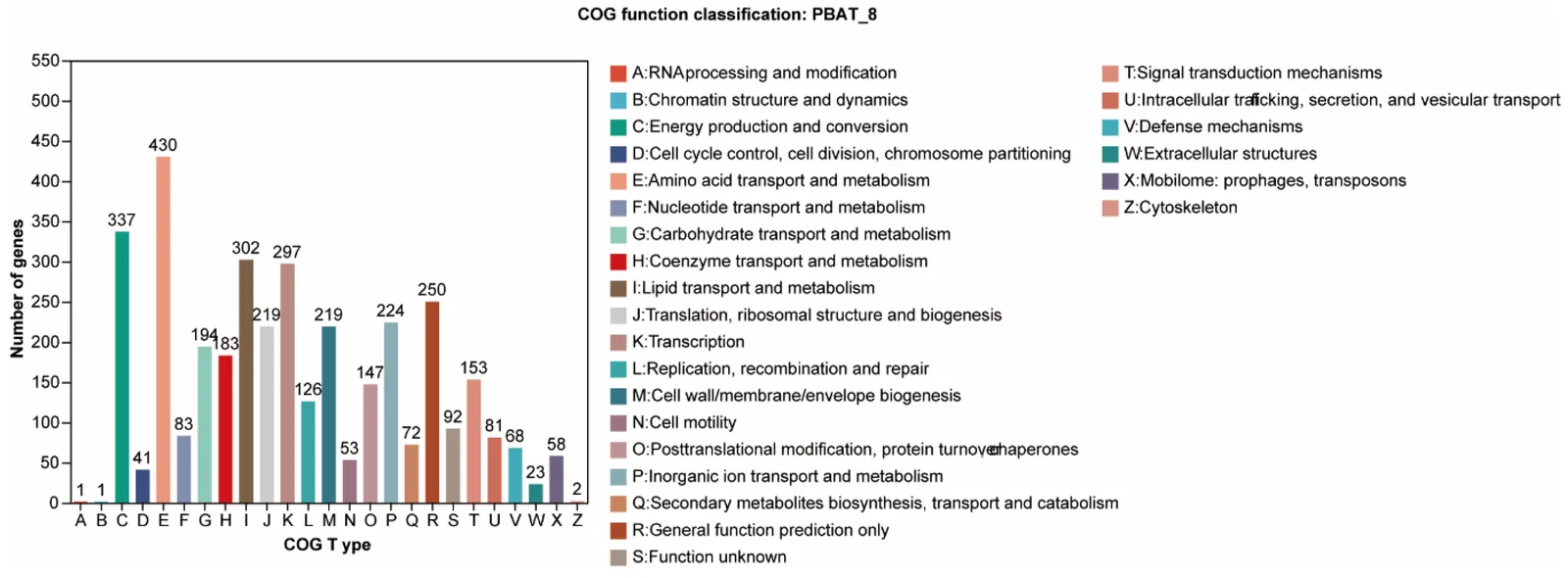
COG functional classification of genes in the PBAT-8 genome. Letters correspond to standard COG functional categories, bars show the number of genes assigned to each category, and bar colors correspond to the COG categories shown in the legend.

**Figure 4:**
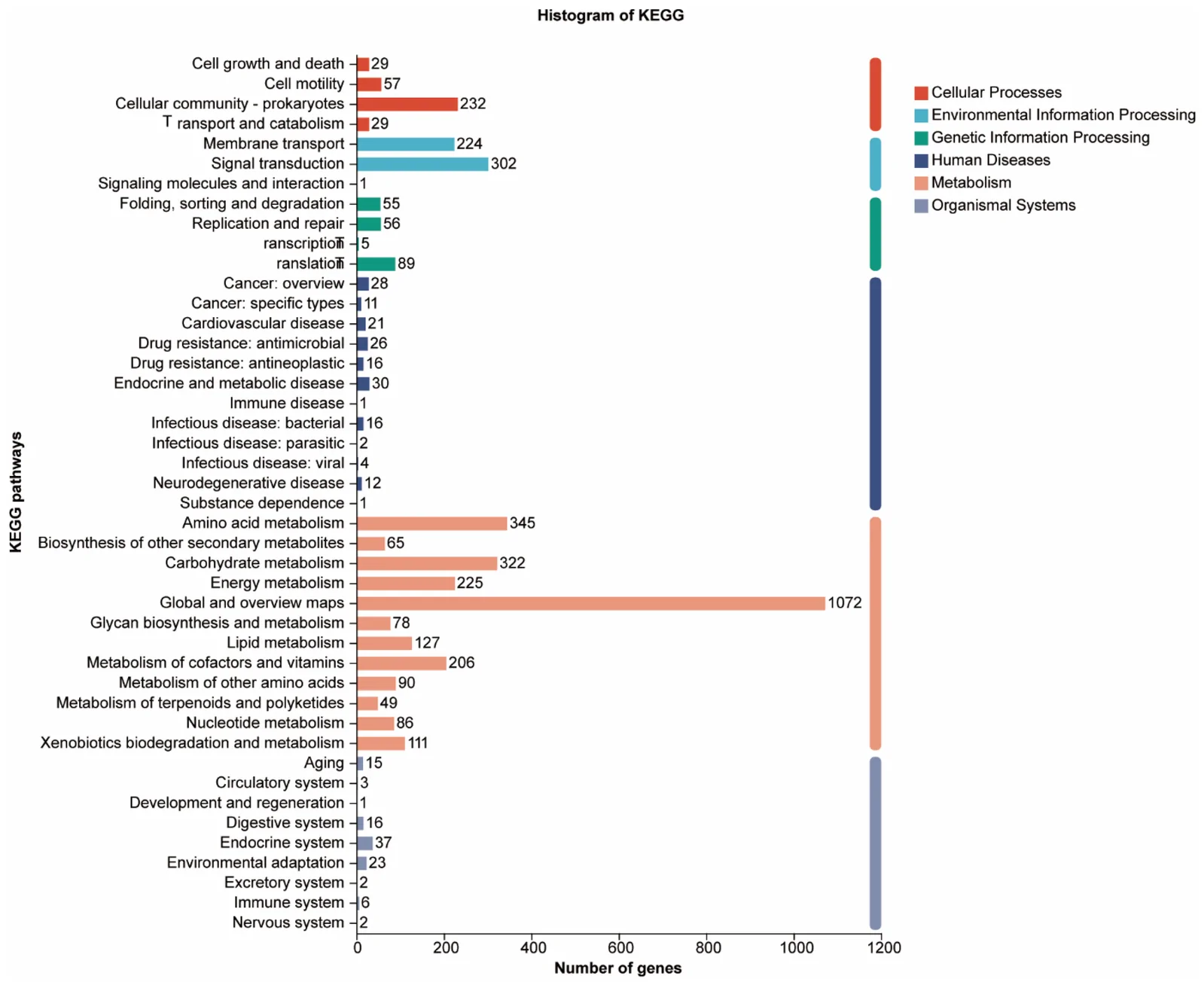
KEGG pathway classification of genes in the PBAT-8 genome. The *x*-axis shows the number of annotated genes, the *y*-axis shows KEGG level-2 pathway categories, and colors indicate KEGG level-1 functional classes.

**Figure 5:**
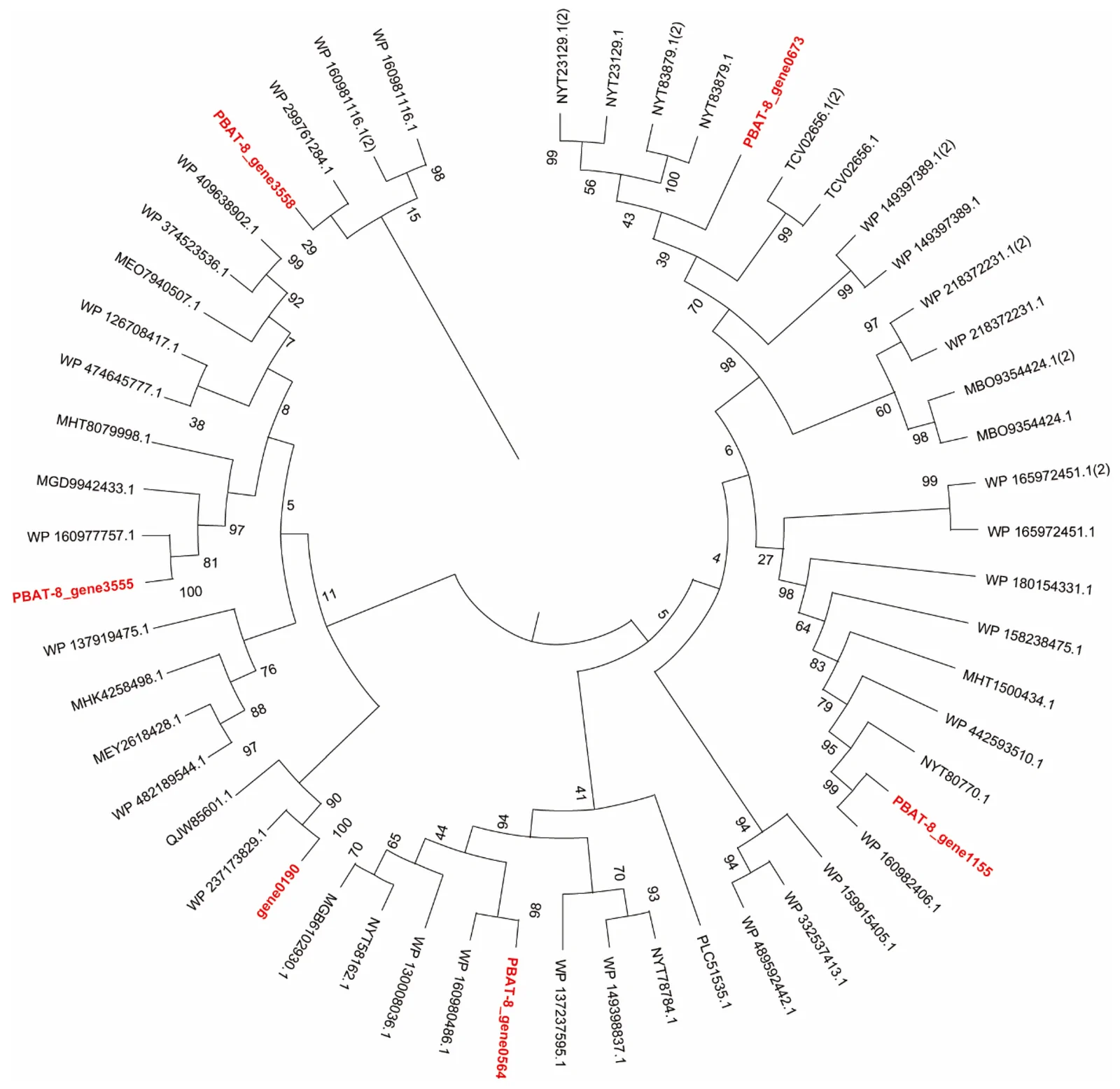
Maximum-likelihood phylogenetic tree of six candidate hydrolase-related proteins from *Paracandidimonas lactea* PBAT-8 and homologous reference proteins retrieved from NCBI and Swiss-Prot. Protein sequences were aligned using MAFFT, and the tree was constructed using IQ-TREE with 1000 bootstrap replicates. Accession numbers are shown at the branch tips, and the six PBAT-8 proteins are highlighted in red.

**Table 1:** General genomic characteristics of *Paracandidimonas lactea* PBAT-8.

Metric	Value
Strain name	PBAT-8
Number of chromosomes	1
Genome size (bp)	4,058,150
Illumina sequencing depth (×)	267.7
PacBio HiFi sequencing depth (×)	67.22
Coverage based on K-mer analysis (%)	96.5%
Coverage based on reads mapping (Next-Generation Sequencing) (%)	100%
Coverage based on reads mapping (Third-Generation Sequencing) (%)	100%
GC Content (%)	61.3
Number of plasmids	0

**Table 2:** Gene prediction statistics for the PBAT-8 genome.

Type	Value
Number of Coding Genes	3658
Total Length of Coding Genes (bp)	3,656,508
Average Gene Length (bp)	999.59
Gene Density (genes/kb)	0.90
GC Content of Coding Regions (%)	61.77
Percentage of Coding Regions in Genome (%)	90.10
Length of Intergenic Regions (bp)	401,642
GC Content of Intergenic Regions (%)	56.98
Percentage of Intergenic Regions in Genome (%)	9.90
Number of rRNAs	9
Number of tRNAs	55
Number of sRNAs	21
Number of Housekeeping Genes	31

**Table 3:** Candidate hydrolase-related genes selected for phylogenetic analysis.

Gene	EC Number	Nr_ID	Nr_Description
gene0190	EC:3.1.1.24	WP_160977175.1	alpha/beta fold hydrolase
gene0673	EC:3.1.1.24	WP_160981116.1	alpha/beta hydrolase
gene1155	EC:3.5.1.106	MYN15008.1	alpha/beta fold hydrolase
gene3555	EC:3.1.1.24/EC:4.1.1.44	WP_160977757.1	alpha/beta fold hydrolase
gene3558	EC:3.1.1.24	WP_202411626.1	alpha/beta hydrolase
gene0564	-	WP_160980486.1	dienelactone hydrolase family protein

## Data Availability

The BioProject record for Paracandidimonas lactea PBAT-8 will be publicly accessible in NCBI under accession PRJNA1497611 no later than publication.

## Supplementary Materials

The following supporting information can be downloaded at: https://www.mdpi.com/article/10.3390/toxics14100847/s1, Figure S1: Representative SEM images of PBAT film surfaces after 180 days of incubation.
