# Supplementary material for: Complete Genome Analysis of Paracandidimonas lactea PBAT-8 Reveals Candidate Functions Associated with PBAT Transformation
Source: Toxics. 2026 Sep 23;14(10):847. doi: 10.3390/toxics14100847 (PMC13709854; doi:10.3390/toxics14100847)
Supplement: Supplementary file 1 [file toxics-14-00847-s001.zip › toxics-4510454-supplementary.pdf]

Supplementary Materials

Complete Genome Analysis of *Paracandidimonas lactea* PBAT-8 Reveals Candidate Functions Associated with PBAT Transformation

Xingyu Lin[1,2], Yanshuo Pan[2], Yankun Zhang[1], Chen Tu[2,3*], Ying Liu[2,3], Yujuan Huang[2], Shiqi Ma[2], Dan Wang[2,3], Jing Wei[4], Yongming Luo[2,3], Xiaoping Diao[1,5*]

1. Ministry of Education Key Laboratory for Ecology of Tropical Islands, Key Laboratory of Tropical Animal and Plant Ecology of Hainan Province, College of Life Sciences, Hainan Normal University, Haikou 571158, China.
2. State Key Laboratory of Soil & Sustainable Agriculture, Institute of Soil Science, Chinese Academy of Sciences, Nanjing 211135, China.
3. University of Chinese Academy of Sciences, Beijing 100049, China.
4. Nanjing Institute of Environmental Sciences, Ministry of Ecology and Environment, Nanjing 210042, China.
5. State Key Laboratory of Marine Resource Utilization in South China Sea, Hainan University, Haikou 570228, China.

* Correspondence: Chen Tu, ctu@issas.ac.cn; Xiaoping Diao, diaoxip@hainanu.edu.cn

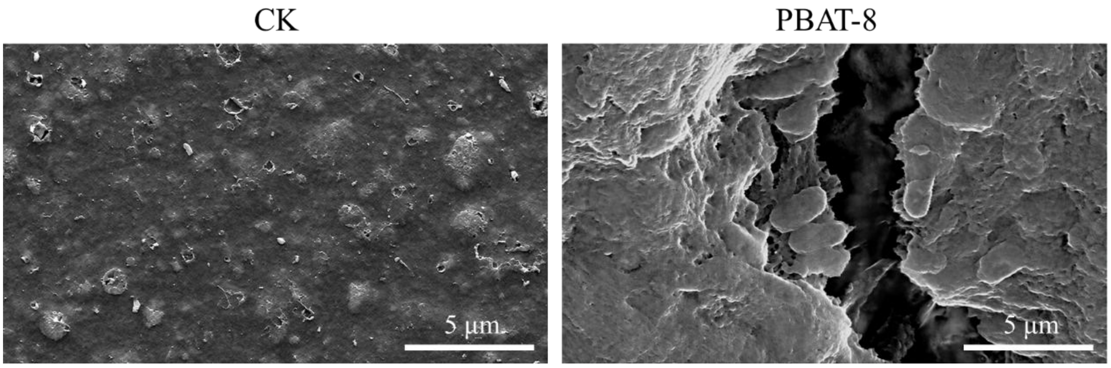

Figure S1. Representative SEM images of PBAT film surfaces after 180 days of incubation. PBAT films were incubated in minimal salt medium at 30°C with shaking at 180 rpm. CK, uninoculated control; PBAT-8, PBAT film incubated with *Paracandidimonas lactea* PBAT-8. Scale bars are shown in the images.
